# Formation of Geopolymers Using Sodium Silicate Solution and Aluminum Orthophosphate

**DOI:** 10.3390/ma13184202

**Published:** 2020-09-21

**Authors:** Stephan Partschefeld, Torben Wiegand, Frank Bellmann, Andrea Osburg

**Affiliations:** 1 Chair of Construction Chemistry and Polymer Materials, F.A. Finger Institute of Building Materials Science, Bauhaus-Universität Weimar, Weimar 99423, Germany; torben.wiegand@uni-weimar.de (T.W.); andrea.osburg@uni-weimar.de (A.O.); 2 Chair of Building Materials, F.A. Finger Institute of Building Materials Science, Bauhaus-Universität Weimar, Weimar 99423, Germany; frank.bellmann@uni-weimar.de

**Keywords:** geopolymer, berlinite, sodium silicate solution, aluminum orthophosphate, alumosilicate, sodium phosphate hydrate, XRD, NMR, calorimetry

## Abstract

This paper reports the formation and structure of fast setting geopolymers activated by using three sodium silicate solutions with different modules (1.6, 2.0 and 2.4) and a berlinite-type aluminum orthophosphate. By varying the concentration of the aluminum orthophosphate, different Si/Al-ratios were established (6, 3 and 2). Reaction kinetics of binders were determined by isothermal calorimetric measurements at 20 °C. X-ray diffraction analysis as well as nuclear magnetic resonance (NMR) measurements were performed on binders to determine differences in structure by varying the alkalinity of the sodium silicate solutions and the Si/Al-ratio. The calorimetric results indicated that the higher the alkalinity of the sodium silicate solution, the higher the solubility and degree of conversion of the aluminum orthophosphate. The results of X-ray diffraction and Rietveldt analysis, as well as the NMR measurements, confirmed the assumption of the calorimetric experiments that first the aluminum orthophosphate was dissolved and then a polycondensation to an amorphous aluminosilicate network occurred. The different amounts of amorphous phases formed as a function of the alkalinity of the sodium silicate solution, indicate that tetrahydroxoaluminate species were formed during the dissolution of the aluminum orthophosphate, which reduce the pH value. This led to no further dissolution of the aluminum orthophosphate, which remained unreacted.

## 1. Introduction

Novel binder systems are being developed and researched worldwide to minimize CO_2_-emissions in the construction industry, especially in the production of cement [1]. Clays are alternative raw materials that are available in large quantities worldwide. They are independent of other branches of industry and can be activated by moderate thermal treatment at 500–800 °C. Such a famous material, which is used in many researches is metakaolin. By addition of an alkaline activator like alkali hydroxide solution or alkali silicate solution, the metakaolin dissolves and the silicon-and aluminum-species combine in a polycondensation reaction to an X-ray amorphous alumosilicate network [2]. The alkalis are firmly incorporated into the structure of the formed geopolymers [3]. The solidification time of the geopolymers takes several hours to days, depending on the composition of the aluminum silicate sources and the concentration of the activator. Singh and Middendorf investigated whether geopolymers are an alternative to OPC. The authors examined different alumino silicate source materials like calcined clays, fly ash, rice husk ash, slag, waste glass and different alkaline activators like sodium silicate, NaOH, KOH and Ca(OH)_2_. It was found that by optimizing the curing temperature, alkali concentrations, additives and Na_2_O/SiO_2_ ratio, geopolymer cements with high mechanical and durability properties can be produced [4]. Gharzouni et al. investigated the effect of reactivity of alkaline solution and metakaolin on geopolymer formation. It was determined that the reactivity of the alkaline solution governs the polycondensation reaction of geopolymers. The reactivity of metakaolin and the depolymerization of the alkaline solution are key parameters, that control the polycondensation rate and the compressive strength of geopolymer materials [5]. Another route to form geopolymers is the activation of a calcined clay by acid solution especially high concentrated phosphoric acid [6]. Celeriere et al. investigated the properties of phosphoric acid based geopolymers made from different reactive types of metakaolin. It was found that the samples with the more reactive metakaolin had a rougher and more porous microstructure. The samples with the less reactive metakaolin showed a smoother and denser microstructure [7]. Tchakouté et al. investigated the influence of different concentrations of phosphoric acid on the properties of phosphate-based geopolymers formed by addition to metakaolin. It could be shown, that by increasing concentration of phosphoric acid the strength of the test specimens increased. It was also found that berlinite was formed, which was finely dispersed in the geopolymer matrix and contributed to the increase in strength [8]. Tchakouté et al. also conducted comparative studies between water glass activated and phosphoric acid activated metakaolin based geopolymers. It was shown that both geopolymer binders had a homogeneous microstructure and the phosphoric acid activated binder formed crystalline berlinite, which served as a matrix filler [9]. Douiri et al. investigated the thermal and dielectric properties of phosphoric acid based geopolymers in dependence on the SiO_2_/H_3_PO_4_ ratio. It could be shown, that by increasing the concentration of the phosphoric acid the samples become more amorphous, which leads to a change in their thermal behavior. Furthermore, it was determined that the permittivity, the dielectric loss and the electrical conductivity strongly depend on the H_3_PO_4_ concentration [10]. Detailed investigations by Douiri et al. on the dielectric properties of metakaolin and phosphoric acid based geopolymers showed, that a significant decrease in dielectric parameters occurs in 28-day old samples heated to high temperatures [11]. Liu et al. investigated the phase evolution of geopolymers prepared by an Al_2_O_3_-2SiO_2_ powder of sol-gel synthesis and phosphoric acid at elevated temperatures. The produced geopolymer showed a high thermal stability, with no sign of melting up to 1550 °C. The onset of crystallization starts at 900 °C [12]. The kinetic of acid based geopolymers by the use of different particle size of a calcined illito-kaolinitic clay was studied by Louati et al. The results showed that the fineness of the used precursor has a significant influence on geopolymerization kinetics and the mechanical properties of the product. The authors also proposed a three-step mechanism of geopolymerization [13]. Wang et al. investigated the formation mechanism and thermal stability of a chemosynthetic phosphate based geopolymer. At 28 days, the hardened geopolymer paste with an Al/P molar ratio of 1.0 reached a compressive strength of 31 MPa. An amorphous structure of SiO_2_·Al_2_O_3_·P_2_O_5_·*n*·H_2_O and a crystalline phase of aluminum hydrogen phosphate were detected. At elevated temperatures, a phase transition to berlinite and a silicon containing phase was found [14]. Zribi et al. investigated the effect of curing temperature on the properties of phosphate based geopolymers, that is, the structural and mechanical properties [15]. Bothe and Brown studied the formation of AlPO_4_ using Al_2_O_3_ with high specific surface area and low crystallinity. The aluminum components were diluted in 33.3 wt.% H_3_PO_4_ based on the amount needed to form stoichiometric AlPO_4_. Thermal treatment of the formed hydrated aluminum orthophosphates from 113 °C to 133 °C led to anhydrous AlPO_4_-geopolymers [16]. Perera et al. investigated the geopolymerization using metakaolin and phosphoric acid milieu. A material with a high compressive strength in the range of 140–146 MPa was formed which had twice the value of the same geopolymer created under alkaline conditions [17]. ^29^Si nuclear magnetic resonance (NMR) investigations were performed by Louati et al. with the same conditions described by Perera [13]. Depending on the Si/P molar ratio a Si-O-P-P-Al-OH sequences were found. Wagh described chemically bonded phosphate ceramics as a new class of geopolymers [18]. Most of the previous investigations were based on the acid activation of aluminate sources by phosphoric acid, which led to berlinite-type geopolymers. In this study a new way to synthesize a room temperature fast setting geopolymer, using aluminum orthophosphate (AlPO_4_, berlinite-type) as aluminate source and sodium silicate solutions is shown.

## 2. Materials and Methods 

### 2.1. Materials

In this study, a basic sodium silicate solution Betol 39T^®^ (Wöllner GmbH, Ludwigshafen, Germany) was used. In preliminary tests, it was found that the solidification time of the geopolymers depends directly on the alkalinity of the sodium silicate solution. Therefore, the sodium silicate solution was modified by the addition of sodium hydroxide pellets (Carl Roth GmbH + Co. KG, Karlsruhe, Germany; purity ≥ 98 wt.%) whereby three sodium silicate solutions (WG) with modules 2.4, 2.0 and 1.6 were produced. The molar modules, water content, dynamic viscosity and the surface tension characteristics are shown in Table 1. 

**Figure 1:**
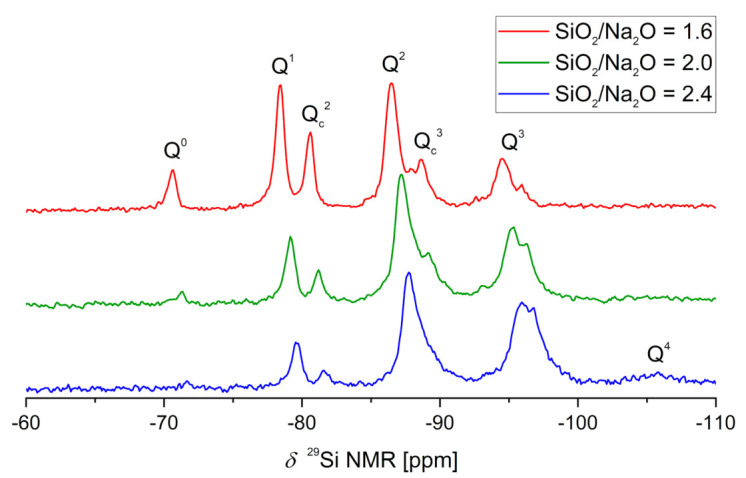
Static ^29^Si nuclear magnetic resonance (NMR) spectra of used silica solutions.

The starting materials were also characterized by NMR spectroscopy. The NMR conditions are given in Section 2.3 (NMR measurements). Figure 1 shows the static ^29^Si NMR spectra of used silica solutions. The labelling of the signals (Q^n^) provides information about the structural units of the silica species depending on the sodium content. Since the silicate ion can form up to four bonds, *n* can achieve values between 0 and 4 depending of the degree of condensation [19,20]. As expected, the highest amount of isolated silicon tetrahedrons (Q^0^) in solution can be observed in the solution with the lowest SiO_2_/Na_2_O molar ratio. Associated with this, no signals of Q^4^ groups are detectable. The figure also shows a typical slight downfield shift of the signals of every silica species with higher sodium contents in solution [19]. 

**Figure 2:**
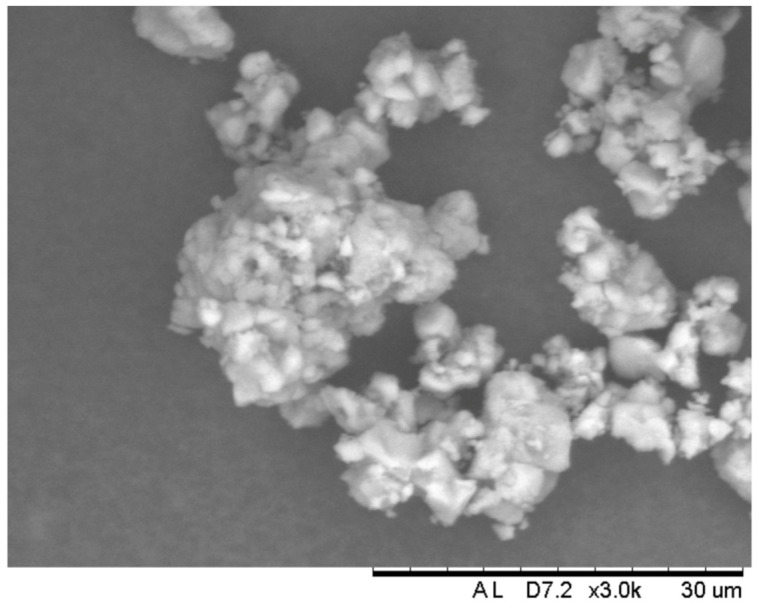
Scanning electron microscopy (SEM) image of the used aluminum orthophosphate.

A berlinite-type aluminum orthophosphate (Zschimmer & Schwarz GmbH & Co KG, Lahnstein, Germany) was used as the aluminum source. The chemical and mineralogical composition of this compound is shown in Table 2. Figure 2 shows a SEM image (Scanning Electron Microscope, TM 3000 Tabletop microscope, Hitachi, Tokio, Japan) of the used aluminum orthophosphate to illustrate the crystalline morphology of the starting material.

**Figure 3:**
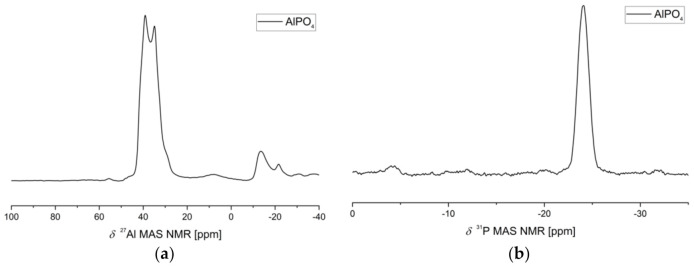
^27^Al MAS NMR spectrum (**a**) and the ^31^P MAS NMR spectrum (**b**) of the used AlPO_4_.

In order to verify the completeness of the conversion of this starting material to geopolymer, NMR spectra of the used berlinite were recorded. Figure 3 shows the ^27^Al MAS NMR spectrum (Figure 3a) and the ^31^P MAS NMR spectrum (Figure 3b) of the used AlPO_4_.

The two signals in ^27^Al MAS NMR spectrum at about 39 and 35 ppm correspond very well with the literature values for tetrahedral coordinated AlPO_4_. In contrast, the less intense signals at −13 and −21 ppm can be attributed to the presence of octahedral coordinated aluminum species [21].

The ^31^P MAS NMR spectrum shows a single signal at −24 ppm, which corresponds very well with the literature values for berlinite [20].

### 2.2. Methods

The aluminum orthophosphate was characterized by particle size analysis (Laser particle analyzer, LS 230, Coulter, Indianapolis, IN, USA) and measurements of BET-surface (BET Analyzer, Coulter SA 3100). The mineralogical and chemical analysis were performed by X-ray phase analysis (X-ray diffractometer Seifert, New York, NY, USA, XRD 3003 TT with Euler cradle and X-Y table and Rietveldt analysis) and ICP-OES (inductively coupled plasma optical emission Spectroscope, Aktiva M, Horiba, Kyoto, Japan). Table 2 shows the material parameters of the used aluminum orthophosphate. 

The viscosity and the surface tension of sodium silicate solutions depend on the alkali content. Typically, sodium silicate solutions show a decrease in viscosity with increasing alkali content, up to a limit concentration. With further increase of the alkali content the viscosity rises again. However, the viscosity and the surface tension of water glasses are important parameters for workability of geopolymer binders. Therefore, the produced sodium silicate solutions were also characterized by rheological measurements using a rotation viscometer (Rheotec^®^ Brookefield DV III-ultra, Middleboro, MA, USA) with SC-4 18 spindle. The dynamic viscosity measurements were performed at constant rotation speed of 60 rpm. Furthermore, the surface tension of the sodium silicate solutions in dependence on the module were measured with a tensiometer (K 100, Krüss, Hamburg, Germany) using the Pt-plate method. The Wilhelmy Equation (1) was applied in order to obtain the surface tension from the recorded force.

(1) $$\sigma =\frac{F}{L\times \cos\theta }\;\sigma :\mathrm{surface}\;\mathrm{tension};F:\mathrm{force};L:\mathrm{wetted}\;\mathrm{length};\cos\theta :\mathrm{contact}\;\mathrm{angle}$$

Calorimetric measurements to determine the reaction kinetics of the geopolymer binders depend on the concentrations of both aluminum orthophosphate and alkali-silicate solution. The measurements were performed with an isothermal calorimeter (mc cal^®^, C3-Prozesstechnik, Gieboldehausen, Germany) at 20 °C for 24 h. Mixing of alkali-silicate solution and aluminum orthophosphate took place inside the calorimeter by means of an electric stirrer. Each calorimetric experiment was performed threefold and a mean heat flow curve was determined. 

The geopolymer binders were characterized by X-ray phase analysis using annealed crystalline ZnO (Particle size distribution: d_10_ = 0.61 µm; d_50_ = 1.27 µm; d_90_ = 2.17 µm) to determine the amount of amorphous phase. Therefore, geopolymer binders were ground to an average particle size of under 28 µm. Each sample was wet ground and homogenized with 10% internal zirconium and 10 mL isopropanol for 1 min with a vibrating rod mill (MC Crone, MicroNising, London, UK) using corundum grinding media. The samples were dried for 3 h in a drying oven at 40 °C and placed in the sample holders for X-ray phase analysis. The amount of crystalline and amorphous phases was calculated by Rietveldt method using the program AutoQuan^®^, Boston, MA, USA.

### 2.3. NMR Measurements

NMR measurements were performed on an Oxford wide bore magnet (11.7 T) attached to a Bruker Avance III console.

#### 2.3.1. ^27^Al NMR Measurements

A Bruker triple resonance probe head for 3.2 mm (O.D.) zirconia rotors was used for ^27^Al NMR solid state magic angle spinning (MAS) NMR spectroscopy. The single-pulse spectra were collected at a MAS spinning rate of 10 kHz employing the following conditions: pulses width 0.5 µs at 200 W (RF field strength = 60 kHz), relaxation delay time 2.0 s, 1024 scans, ^1^H decoupling by TPPM15 at 100 W (RF field strength = 76 kHz) with PCPD2 = 6.5 µs. An exponential decay function (LB 100) was applied to improve the signal to noise ratio. The chemical shifts were referenced to a 1.0 M aqueous solution of AlCl_3_.

#### 2.3.2. ^29^Si MAS and Static NMR

An *NMR Service Erfurt* triple resonance probe head for 7 mm (O.D.) zirconia rotors was used for ^29^Si MAS NMR and static NMR spectroscopy. The single pulse spectra of the solid samples were collected at a MAS spinning rate of 4 kHz, with 8 µs pulses at 100 W (90° tip angle, RF field strength = 32 kHz) and a recycle delay time of 1200 s. 72 scans were accumulated with 1H decoupling using TPPM15 at 200 W (RF field strength = 43 kHz) with PCPD2 = 11.3 µs. An exponential decay function (LB 50) was applied to improve the signal to noise ratio. The liquid samples were analyzed using related conditions but without MAS (256 scans, 60 s recycle delay time, 8 µs pulse width, 32 kHz RF field strength, 1H decoupling using TPPM15). The zirconia rotor was protected against the highly basic solutions using a KEL-F insert. All chemical shift data were reported relative to external tetramethylsilane (TMS).

#### 2.3.3. ^31^P NMR Measurements

A Bruker triple resonance probe head for 3.2 mm (O.D.) zirconia rotors was used for ^31^P NMR MAS NMR spectroscopy. The single pulse spectra were collected at a MAS spinning rate of 4 kHz, with 12 µs pulses at 150 W (90° tip angle, RF field strength = 21 kHz) and a recycle delay time of 60 s. 128 scans were accumulated with 1H decoupling using TPPM15 at 15 W (rf field strength = 17 kHz) with PCPD2 = 20 µs. An exponential decay function (LB 20) was applied to improve the signal to noise ratio. ^31^P NMR chemical shifts were referenced to 85% H_3_PO_4_ using a solid sample of Li_3_PO_4_ as a secondary reference (δ_so_ = 10.8 ppm [22,23]).

## 3. Results and Discussion

### 3.1. Calorimetric Studies

**Figure 4:**
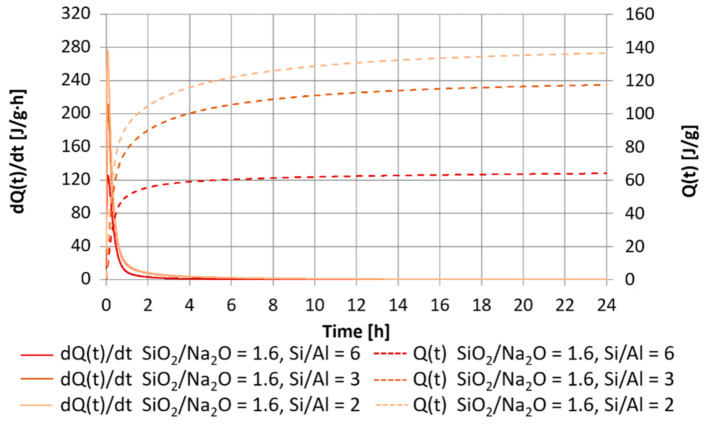
Calorimetric measurements of the reaction kinetics of sodium silicate solution (module: 1.6) and different Si/Al-ratios of 2, 3 and 6.

**Figure 5:**
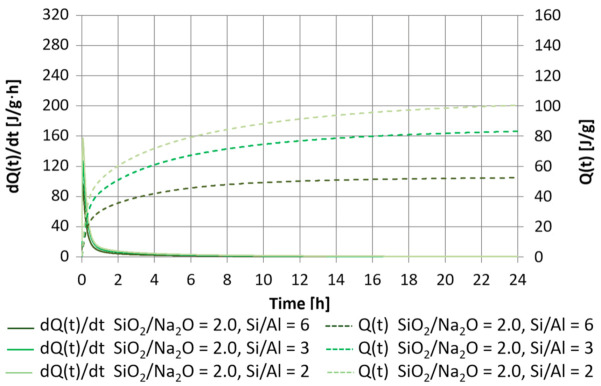
Calorimetric measurements of the reaction kinetics of sodium silicate solution (module: 2.0) and different Si/Al-ratios of 2, 3 and 6.

**Figure 6:**
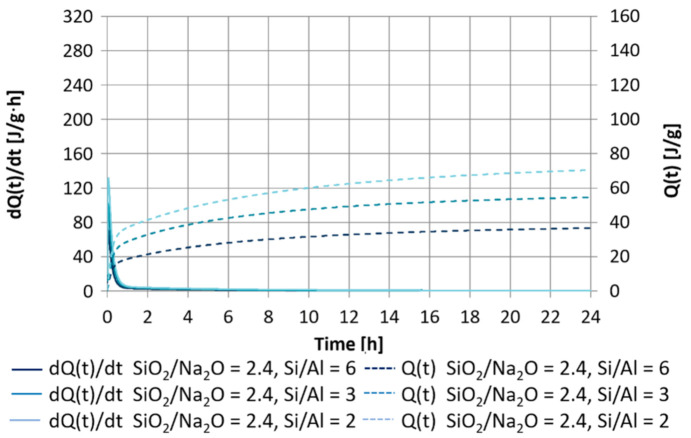
Calorimetric measurements of the reaction kinetics of sodium silicate solution (module: 2.4) and different Si/Al-ratios of 2, 3 and 6.

Calorimetric experiments were performed to determine the reaction mechanism of aluminum orthophosphate and sodium silicate solutions depending on their alkalinity. Figure 4 shows the heat flow and the total heat release of the sodium silicate solutions (module: 1.6) mixed with 10 wt.% (Si/Al-ratio = 6), 20 wt.% (Si/Al-ratio = 3) and 30 wt.% (Si/Al-ratio = 2) of aluminum orthophosphate. It is evident, that the heat flow increased with increasing concentration of aluminum orthophosphates. The first heat release, which corresponded to wetting and dissolution of the aluminum orthophosphate, increased by increasing its concentration. In addition, after the first 30 min a peak broadening occurred, which corresponded to a polycondensation process of the dissolved aluminum and silicon species to an alumosilicate network. The total heat release increased from 64 J/g (Si/Al = 6) to 117 J/g (Si/Al = 3) during the first 24 h, when the aluminum orthophosphate concentration was doubled. A further increase of the aluminum orthophosphate concentration to a Si/Al-ratio of 2 led to a much smaller increase of heat release of 136 J/g. This indicates that the aluminum orthophosphate was not completely dissolved. Accordingly, increasing the concentration of aluminum orthophosphate from Si/Al-ratio of 3 to Si/Al-ratio of 2, did not lead to a proportional increase in the degree of conversion. Thus, a certain proportion of unreacted aluminum orthophosphate was present in the alumosilicate network. Figure 5 shows the heat flow and the total heat release by using the sodium silicate solutions with module 2.0. The same tendencies in heat flow and total heat release could be observed. In contrast to the sodium silicate solution with module 1.6, the total amount of heat release was significantly lower, especially at the concentrations of 20 wt.% (83 J/g) and 30 wt.% (100 J/g) of aluminum orthophosphate. When using the sodium silicate solution with module 2.4 (Figure 6) the tendency of non-proportional conversion of the aluminum orthophosphate depending on its concentration was less pronounced. However, in comparison to the sodium silicate solution with module 1.6, the total heat release was lower by a factor of approx. 2 for all added amounts of aluminum orthophosphate. Nevertheless, the calorimetric results indicate that the higher the alkalinity of the sodium silicate solution, the higher the solubility and degree of conversion of the aluminum orthophosphate.

### 3.2. XRD-Analysis

**Figure 7:**
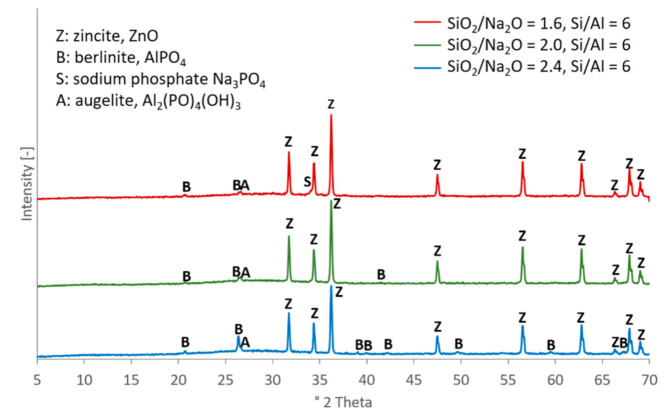
Phase composition of the formed geopolymer binders using a Si/Al-ratio of 6 and different sodium silicate solution (SiO*_2_*/Na*_2_*O = 1.6, 2.0 and 2.4).

The binders produced for the calorimetric investigations were examined by X-ray powder diffraction regarding their phase composition. Especially the number of amorphous phases as well as the content of unreacted aluminum orthophosphate were determined. In addition, it was examined whether crystalline phases are formed. Figure 7 shows the X-ray diffraction diagram of geopolymer binders with a Si/Al-ratio of 6 and the three sodium silicate solutions. Small reflexes of berlinite and augelite were detected. Sodium phosphate was only detected for the sample with sodium silicate solution with low module (1.6). 

**Figure 8:**
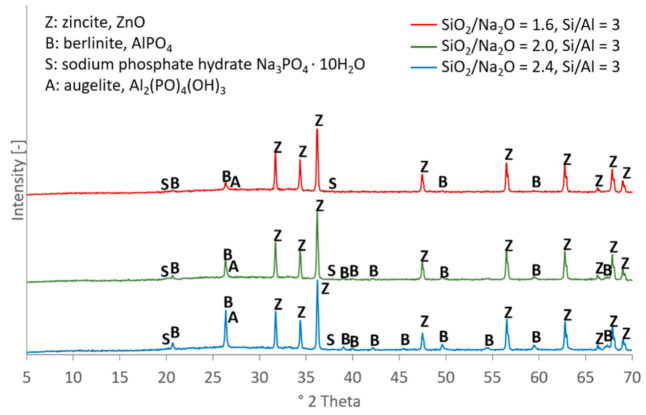
Phase composition of the formed geopolymer binders using a Si/Al-ratio of 3 and different sodium silicate solution (SiO*_2_*/Na*_2_*O = 1.6, 2.0 and 2.4).

Figure 8 shows the X-ray diffraction diagram of binders, which were produced with a Si/Al-ratio of 3 and the three sodium silicate solutions. Like the measurements of binders with Si/Al-ratio of 6, small reflexes of berlinite and augelite were detected. In contrast small reflexes of sodium phosphate–10–hydrate was observed for all binders. The amount of this phase varied from approx. 2 wt.% (WG-1.6) to 3.2 wt.% (WG-2.0) and 2.9 wt.% (WG-2.4). The amount of amorphous phase followed the same order for binders with Si/Al-ratio of 6. The highest amount of amorphous phase of 94.6 wt.% was detected for the sodium silicate solution with module 1.6. By decreasing alkalinity of the sodium silicate solution, the amount of amorphous phase decreased to 90 wt.% (WG-2.0) and 87 wt.% (WG-2.4). In the same manner the amount of unreacted berlinite increased from 1.5 wt.% (WG-1.6) to 4.5 wt.% (WG-2.0) and 9.8 wt.% (WG-2.4). The amount of augelite increased slightly from 1.9 wt.% to 2.1 wt.% by increasing the alkalinity of sodium silicate solutions. Furthermore, by increasing the amount of added aluminum orthophosphate, the amorphous phase decreased and the amount of berlinite increased at all sodium silicate solutions. 

**Figure 9:**
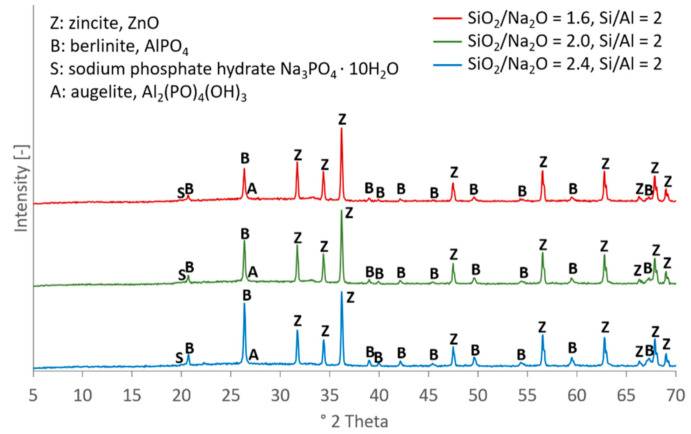
Phase composition of the formed geopolymer using a Si/Al-ratio of 2 and different sodium silicate solution (SiO*_2_*/Na*_2_*O = 1.6, 2.0 and 2.4).

Figure 9 shows the X-ray diffraction diagram of geopolymer binders, which were created with a Si/Al-ratio of 2 and the three sodium silicate solutions. Like the diffractograms with Si/Al-ratio of 3, berlinite, augelite and sodium phosphate-10-hydrate were detected. The amorphous phase fraction increased significantly by increasing the alkalinity of the sodium silicate solution from approx. 78 wt.% (WG-2.4) to 84 wt.% (WG-2.0) and 90 wt.% (WG-1.6). In contrast the amount of unreacted berlinite increased the lower the alkalinity of sodium silicate solutions from 6.8 wt.% (WG-1.6) to 10.9 wt.% (WG-2.0) and 16.5 wt.% (WG-2.4). The results of X-ray diffraction and Rietveldt analysis confirmed the assumption of the calorimetric experiments that first the aluminum orthophosphate was dissolved and then a polycondensation to an amorphous aluminosilicate network occurred. The different amounts of amorphous phases formed as a function of the alkalinity of the sodium silicate solution, indicate that tetrahydroxoaluminate species were formed during the dissolution of the aluminum orthophosphate, which reduced the pH value. This led to no further dissolution of the aluminum orthophosphate, which remained unreacted.

Table 3 shows the phase composition of the investigated binders performed by Rietveldt analysis. The amount of amorphous phase in binders with a Si/Al-ratio of 6 was similar for all sodium silicate solutions and varied between 93 and 96 wt.%. The amount of formed augelite varied between 1.8 wt.% (WG-1.6) and 2.4 wt.% (WG-2.4). As proposed in calorimetric experiments, the content of unreacted berlinite increased by decreasing alkalinity of sodium silicate solution from 0.2 wt.% (WG-1.6) to 4 wt.% (WG-2.4). The amount of detected sodium phosphate for the sodium silicate solution with module 1.6 was calculated to 2.8 wt.%. 

### 3.3. NMR-Analysis of Selected Geopolymer Binders

Due to the high amount of amorphous material found by X-ray diffractometric analysis, five hardened binder compositions have been selected for MAS NMR spectroscopic investigations. The chosen binders are given in Table 4.

#### 3.3.1. ^29^Si-NMR

**Figure 10:**
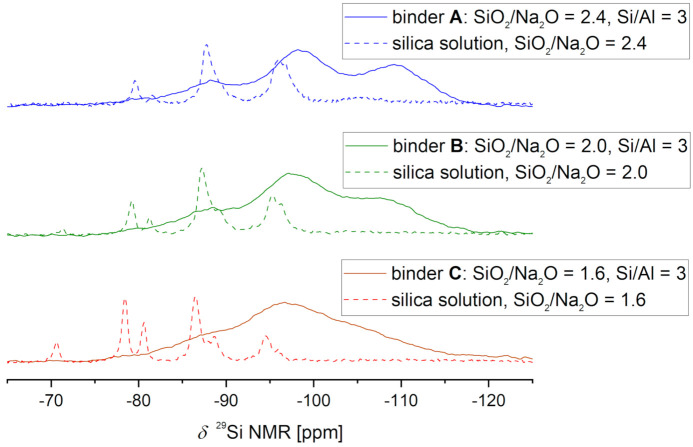
Static ^29^Si NMR of silica solution (SiO_2_/Na_2_O = 2.4, 2.0 and 1.6, dotted lines) and ^29^Si MAS NMR of binders A-C (solid lines), formed by addition of berlinite (Si/Al = 3) to these solutions.

Figure 10 shows the ^29^Si NMR spectra (static NMR) of silica solutions (dotted lines) with SiO_2_/Na_2_O-ratios of 2.4, 2.0 and 1.6 as well as the ^29^Si MAS NMR spectra of binders A, B and C (solid lines) formed by addition of berlinite (Si/Al = 3) to the silica solutions.

The broad signals indicate a largely amorphous structure of the solid, as was already evident from XRD analysis. As expected, no Q^0^ unit was observed in the solid binders. It is also apparent that almost no Q^1^ units were built into formed solids. The position of the Q^4^ signals maxima of the solid samples were high field shifted compared to signals of the silica solution which is a typical observation for silicate structural units [24]. The ^29^Si MAS NMR signals of binder B were in the same range as for binder A but a slightly lower amount of Q^4^ units in the solid and an even more disordered structure of the material resulting in less distinct signal maxima could be observed. This trend of a reduced content of Q^4^ units was continued when the alkali content of the silica solution was further increased, as happened with the preparation of binder C. Binder C exhibited a more disordered Si-O bond environment than the other two compounds because the maxima at Q^2^ and Q^4^ were no longer visible. This agreed with the results of XRD analysis (Table 3).

**Figure 11:**
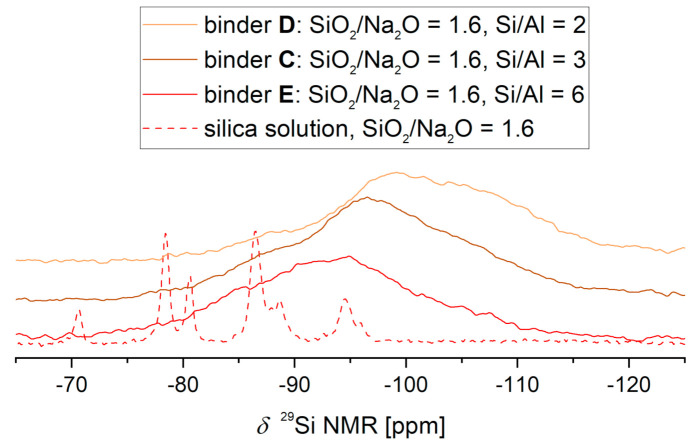
Static ^29^Si NMR of silica solution (SiO_2_/Na_2_O = 1.6, red dotted line) and ^29^Si MAS NMR of binders C-E, formed by addition of berlinite (Si/Al = 2, 3 and 6) to this solution.

Figure 11 shows the ^29^Si MAS NMR spectra of binders C, D and E which were formed by the addition of the silica solution with a SiO_2_/Na_2_O ratio of 1.6.

**Scheme 1:**
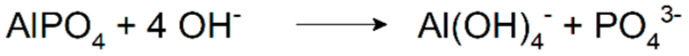
Dissolution reaction of berlinite under alkaline conditions.

It can be clearly seen that binder D (Figure 10, orange line), the binder with the highest aluminum content, also contained the highest proportion of Q^4^ units. This can be attributed to a significant reduction of the pH value due to the dissolution reaction of berlinite (see Scheme 1) and the associated condensation reaction of the silicate ions.

The reduced pH value in turn led to an incomplete dissolution of berlinite, which can also be seen in Table 3. When the Si/Al ratio was increased to 3 (binder C, Figure 10), which corresponds to a reduction of the aluminum content, the pH value did not decrease as much as in the previous samples due to the dissolution reaction. This led to a lower degree of condensation of the silicate ions before the incorporation of the aluminum ions occurred and thus to a lower proportion of Q^4^ units in binder C compared to binder D. This trend continued as the aluminum content was further reduced (binder E, Si/Al = 6). The still high pH value caused an almost complete dissolution of AlPO_4_ (see Table 3) as well as a low degree of condensation of the silicate ions before the aluminate ions were incorporated. This can be clearly seen from the shift in the centroid of the signals in ^29^Si MAS NMR spectrum of binder E towards lower condensed silicate species (downfield shift) in comparison with the spectra of binders C and D. The downfield shift may also have been caused or at least amplified by a higher amount of aluminum atoms directly attached to Si-O-bonds [25].

#### 3.3.2. ^27^Al MAS NMR

**Figure 12:**
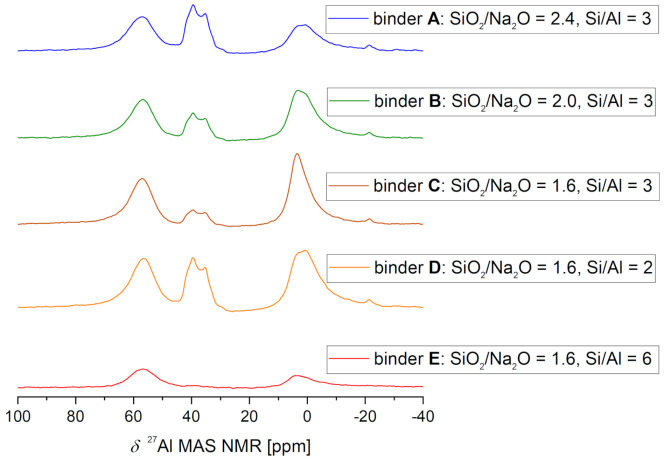
^27^Al MAS NMR spectra of binders A–E.

Figure 12 shows the ^27^Al MAS NMR spectra of binders A–E. All spectra show signals at very similar chemical shifts. 

The signal with the two maxima at about 39 and 35 ppm was caused by AlPO_4_ (berlinite, see also Figure 3a) [20]. As can be seen, the amount of remaining berlinite decreased with increasing pH (binders A–C). With increased addition of AlPO_4_, the proportion of unreacted berlinite increased as expected (binder D). The weak signal at −21 ppm is caused by Al(PO_3_)_3_ impurities in berlinite starting material. Tetrahedral coordinated aluminum atoms, as occurring in geopolymers, were the origin of the intense signal at about 56 ppm. The two superimposed signals at about 2 ppm were produced by octahedral coordinated aluminum atoms, as they also occurred in geopolymer materials [26]. Only binder C showed a single sharper signal at about 3 ppm. This is a clear indication of a more ordered polymeric structure in this sample [27]. Due to the low amount of added berlinite, the NMR signals of binder E were significantly less expressed than in the other samples with the same scaling and the signals of the remaining berlinite were hardly identifiable. 

#### 3.3.3. ^31^P MAS NMR

**Figure 13:**
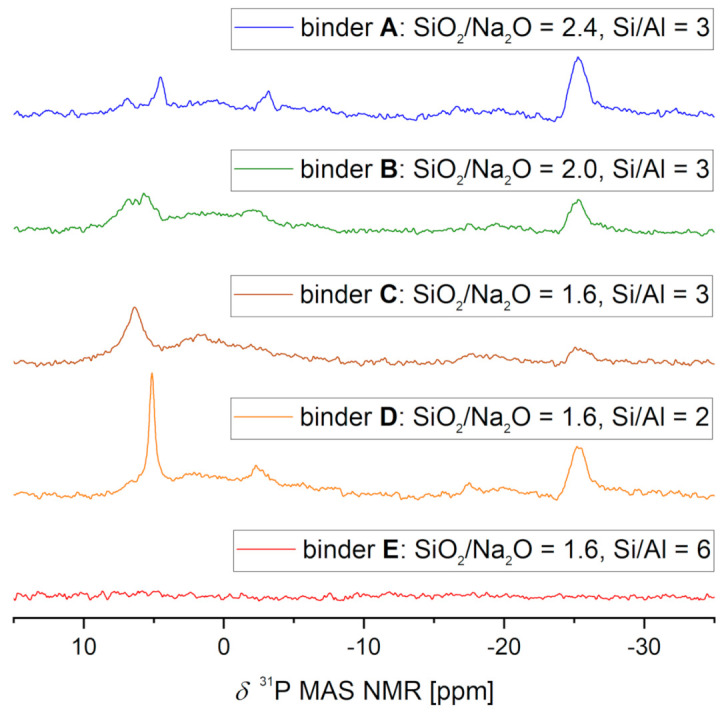
^31^P MAS NMR spectra of binders A*–*E.

Figure 13 shows the ^31^P MAS NMR spectra of binders A to E.

The signal at −24 ppm can be assigned to the unreacted portion of the starting material berlinite (see Figure 3b), which decreased with decreasing SiO_2_/Na_2_O ratio (i.e., increasing pH value; binders A to C). 

As expected, an increase in the berlinite amount at a high pH value (binder D) led to a more intense formation of the berlinite signal. In case of binder E no separated ^31^P NMR signals could be detected due to the markedly reduction of the phosphate content. The remaining phosphorous species seemed to be embedded amorphously in the matrix.

The signals of about 10 to about −2 ppm in the spectra of binders A to D indicate the presence of different sodium phosphate salts. For example, Na_3_PO_4_∙10 H_2_O and Na_3_PO_4_∙12 H_2_O cause a signal at about 8 ppm, Na_2_HPO_4_ causes a signal at about 7 ppm and NaH_2_PO_4_∙H_2_O causes a signal at about 2 ppm [23,28].

## 4. Conclusions

The study dealt with investigations of geopolymer binders prepared from sodium silicate solutions with different alkalinity and a berlinite-type aluminum orthophosphate. By calorimetric investigations it could be shown that the aluminum orthophosphate first was dissolved and tetrahydroxoaluminates were provided, which formed an alumosilicate network by polycondensation with the sodium silicate solution. The alkalinity of the sodium silicate solution played a decisive role in the degree of conversion. The higher the alkalinity of the sodium silicate solution, the more aluminum orthophosphate was dissolved and geopolymer was formed. As assumed in calorimetric experiments, the content of unreacted aluminum orthophosphate increased by decreasing alkalinity of sodium silicate solution independent of the Si/Al-ratio. In this context, the proportion of amorphous phases also decreased with a decreasing Si/Al ratio and pH and less aluminum orthophosphate was dissolved. 

^29^Si MAS NMR measurements showed that the amount of Q^4^ units in the hardened binders decreased with increasing pH value of the silicate solution used. Furthermore, it could be shown that the binder with the highest aluminum content possessed the highest amount of Q^4^ units, which was due to a significant reduction of the pH value by the dissolution of AlPO_4_. By ^27^Al MAS NMR measurements it could be shown that an almost complete dissolution of AlPO_4_ was achieved only with a very small amount of berlinite. With higher pH value the amount of remaining berlinite decreased. ^31^P MAS NMR experiments showed that different sodium phosphate salts were formed during hardening of the binders. The prominent effect of geopolymerization is caused by the berlinite phase of the aluminum orthophosphate, because the Al(PO_3_)_3_ impurities of the starting material is also present in the product, as can be seen in the ^27^Al-NMR at about −21 ppm.

Further research will be conducted on the mechanical properties of the binders for each condition such as compressive and flexural strength. In addition, investigations of the microstructure formation, especially the pore structure, are necessary to obtain information on the durability. A promising field of application for this binder system could be as potential mineral glue for organic and mineral materials. Based on the results of the study, the selection of the starting materials can be stoichiometrically adjusted to achieve optimal conversion rates for the application as mineral adhesive. Due to the direct dependence of the reaction kinetics on the alkalinity of the sodium silicate solution, the solidification time and the degree of conversion can be controlled.

## Figures and Tables

**Table 1:** Parameters of the used sodium silicate solutions.

Sodium Silicate Solutions	SiO_2_/Na_2_O Ratio ^1^ (Molar)	Water Content ^2^ (wt.%)	Dynamic Viscosity (mPa·s)	Surface Tension (mN/m)
Betol 39T^®^	3.3	64.0	97.6	72.9
WG-2.4	2.4	62.7	45.3	78.7
WG-2.0	2.0	61.8	55.7	81.3
WG-1.6	1.6	60.5	90.5	85.9

**^1^** Calculated by chemical analysis by ICO-OES and silicon digestion. **^2^** Calculated after drying at 150 °C.

**Table 2:** Chemical and mineralogical composition of the used aluminum orthophosphate measured by ICP-OES (inductively coupled plasma optical emission spectrometry) and X-ray phase analysis.

Chemical Composition (wt.%)	
Al_2_O_3_	35.9
P_2_O_5_	57.0
SiO_2_	0.6
Na_2_O	0.15
Other Oxides < 0.1 wt.%	0.15
Drying loss	0.9
Annealing loss	5.3
**Mineralogical Composition (wt.%)**	
AlPO_4_ hexagonal	96.74 ± 1.2
Al(PO_3_)_3_	3.26 ± 1.2
**Particle-and Surface Properties**	
BET-surface **^1^** (m^2^/g)	1.925
Particle size d_10_; d_50_; d_90_ (µm)	1.73; 12.95; 94.14

**^1^** Nitrogen gas, p_s_/p_0_ = 0.3, T_BET_ = 105 °C.

**Table 3:** Phase composition of the produced geopolymer binders by Rietveldt-Analysis.

Phase Analysis	SiO_2_/Na_2_O Ratio 1.6 Si/Al Ratio		
	**6**	**3**	**2**
amorphous portion	95.2 ± 0.9	94.64 ± 1.2	89.99 ± 1.3
berlinite	0.23 ± 0.1	1.50 ± 0.5	6.82 ± 0.8
augelite	1.8 ± 0.6	1.89 ± 0.6	1.41 ± 0.8
sodium phosphate	2.78 ± 0.7	-	-
sodium phosphate-10-hydrate	-	1.97 ± 0.7	1.77 ± 0.9
	**SiO_2_/Na_2_O Ratio 2.0** **Si/Al Ratio**		
	**6**	**3**	**2**
amorphous portion	96.57 ± 0.7	90.31 ± 1.8	83.74 ± 2.3
berlinite	1.11 ± 0.3	4.45 ± 0.6	10.89 ± 0.9
augelite	2.32 ± 0.6	2.06 ± 0.7	1.88 ± 0.6
sodium phosphate	-	-	-
sodium phosphate-10-hydrate	-	3.18 ± 1.56	3.49 ± 1.86
	**SiO_2_/Na_2_O Ratio 2.4** **Si/Al Ratio**		
	**6**	**3**	**2**
amorphous portion	93.65 ± 1.0	87.35 ± 2.1	78.37 ± 2.0
berlinite	3.95 ± 0.6	9.75 ± 0.9	16.53 ± 0.9
augelite	2.40 ± 0.7	2.10 ± 0.8	1.61 ± 0.7
sodium phosphate	-	-	-
sodium phosphate-10-hydrate	-	2.92 ± 1.8	2.07 ± 1.7

**Table 4:** Molar SiO_2_/Na_2_O and Si/Al ratios of the selected binders for NMR spectroscopic investigations.

Binder	SiO_2_/Na_2_O Ratio	Si/Al Ratio
A	2.4	3
B	2.0	3
C	1.6	3
D	1.6	2
E	1.6	6

## References

[1] Scrivener K.L., John V.M., Gartner E.M. (2018). Eco-efficient cements: Potential economically viable solutions for a low-CO_2_ cement-based materials industry. Cem. Concr. Res..

[2] Davidovits J. (2015). Geopolymer Chemistry and Applications.

[3] Davidovits J. (1991). Geopolymers. J. Therm. Anal..

[4] Singh N.B., Middendorf B. (2020). Geopolymers as an alternative to Portland cement: An Overview. Constr. Build. Mater..

[5] Gharzouni A., Joussein E., Samet B., Baklouti S., Rossignol S. (2015). Effect of reactivity of alkaline solution and metakaolin on the geopolymer formation. J. Non-Cryst. Solids.

[6] Cao D., Su D., Lu B., Yang Y. (2005). Synthesis and structure characterization of geopolymeric material based on metakaolinite and phosphoric acid. Kuei Suan Jen Hsueh Pao/J. Chin. Ceram. Soc..

[7] Celerier H., Jouin J., Mathivet V., Tessier-Doyen N., Rossignol S. (2018). Composition and properties of phosphoric acid-based geopolymers. J. Non-Cryst. Solids.

[8] Tchakouté H.K., Rüscher C.H., Kamseu E., Andreola F., Leonelli C. (2017). Influence of the molar concentration of phosphoric acid solution on the properties of metakaolin-phosphate-based geopolymer cements. Appl. Clay Sci..

[9] Tchakouté H.K., Rüscher C.H. (2017). Mechanical and microstructural properties of metakaolin-based geopolymer cements from sodium waterglass and phosphoric acid solution as hardeners: A comparative study. Appl. Clay Sci..

[10] Douiri H., Louati S., Baklouti S., Arous M., Fakhfakh Z. (2014). Structural, thermal and dielectric properties of phosphoric acid-based geopolymers with different amounts of H3PO4. Mater. Lett..

[11] Douiri H., Louati S., Baklouti S., Arous M., Fakhfakh Z. (2016). Enhanced dielectric performance of metakaolin–H3PO4 geopolymers. Mater. Lett..

[12] Liu L.-P., Cui X.-M., He Y., Liu S.-D., Gong S.-Y. (2012). The phase evolution of phosphoric acid-based geopolymers at elevated temperatures. Mater. Lett..

[13] Louati S., Baklouti S., Samet B. (2016). Acid based geopolymerization kinetics: Effect of clay particle size. Appl. Clay Sci..

[14] Wang Y.-S., Dai J.-G., Ding Z., Xu W.-T. (2017). Phosphate-based geopolymer: Formation mechanism and thermal stability. Mater. Lett..

[15] Zribi M., Samet B., Baklouti S. (2019). Effect of curing temperature on the synthesis, structure and mechanical properties of phosphate-based geopolymers. J. Non-Cryst. Solids.

[16] Bothe J.V., Brown P.W. (1993). Low-Temperature Formation of Aluminum Orthophosphate. J. Am. Ceram. Soc..

[17] Perera D.S., Hanna J.V., Davis J., Blackford M.G., Latella B.A., Sasaki Y., Vance E.R. (2008). Relative strengths of phosphoric acid-reacted and alkali-reacted metakaolin materials. J. Mater. Sci..

[18] Wagh A. (2012). Chemically Bonded Phosphate Ceramics—A Novel Class of Geopolymers. Ceram. Trans..

[19] Engelhardt L.G., Zeigan D., Jancke H., Wieker W., Hoebbel D. (1975). 29Si-NMR-Spektroskopie an Silicatlösungen. II. Zur Abhängigkeit der Struktur der Silicatanionen in wäßrigen Natriumsilicatlösungen vom Na: Si-Verhältnis. Z. Anorg. Allg. Chem..

[20] Favier A., Habert G., Roussel N., D’Espinose de Lacaillerie J.-B. (2015). A multinuclear static NMR study of geopolymerisation. Cem. Concr. Res..

[21] Müller D., Grunze I., Hallas E., Ladwig G. (1983). Hochfeld-27Al-NMR-Untersuchungen zur Aluminiumkoordination in kristallinen Aluminiumphosphaten. Z. Anorg. Allg. Chem..

[22] Müller D., Jahn E., Ladwig G., Haubenreisser U. (1984). High-resolution solid-state 27Al and 31P NMR: Correlation between chemical shift and mean Al-O-P angle in AlPO_4_ polymorphs. Chem. Phys. Lett..

[23] Turner G.L., Smith K.A., Kirkpatrick R.J., Oldfieldt E. (1986). Structure and cation effects on phosphorus-31 NMR chemical shifts and chemical-shift anisotropies of orthophosphates. J. Magn. Reason..

[24] Lippmaa E., Maegi M., Samoson A., Engelhardt G., Grimmer A.R. (1980). Structural studies of silicates by solid-state high-resolution silicon-29 NMR. J. Am. Chem. Soc..

[25] Engelhardt G., Hoebbel D., Tarmak M., Samoson A., Lippmaa E. (1982). Si-29 Nmr Investigations on the Anion Structure of Crystalline Tetramethylammonium-Aluminosilicates and Aluminosilicate Solutions. Z. Anorg. Allg. Chem..

[26] O’Connor S.J., MacKenzie K.J.D. (2010). A new hydroxide-based synthesis method for inorganic polymers. J. Mater. Sci..

[27] Barbosa V.F.F., MacKenzie K.J.D., Thaumaturgo C. (2000). Synthesis and characterisation of materials based on inorganic polymers of alumina and silica: Sodium polysialate polymers. Int. J. Inorg. Mater..

[28] Shigenobu H., Kikuko H. (1989). High-Resolution Solid-State 31P NMR of Alkali Phosphates. Bull. Chem. Soc. Jpn..

